# Researches on the Effects of Bloodletting in Some Inflammatory Diseases, and on the Influence of Tartarized Antimony and Vesication in Pneumonitis

**Published:** 1837-04

**Authors:** 


### ART. XII.

*Researches on the Effects of Bloodletting in some Inflammatory Diseases, and on the Influence of Tartarized Antimony and Vesication in Pneumonitis.* By P. C. A. LOUIS, Physician of the Hospital la Pitié, &c. *Translated by* C. G. PUTNAM, M.D.; *with Preface and Appendix*, by JAMES JACKSON, M.D., Physician of the Massachusetts General Hospital.—*Boston* (*United States*), 1836. 8vo. pp. 171.

WE have already given so full an account of M. Louis' enquiry into the effects of bloodletting on some inflammatory diseases (*British and Foreign Medical Review*, vol. I. p. 397,) that, so far as Dr. Putnam's share of the present work is concerned, the only duty which devolves upon us is to assure the reader that the translation is faithfully and well executed. The preface and appendix are from the pen of Dr. Jackson, of Boston, whose affecting memoir of his son we had also occasion to notice in a former number. The preface is a reprint from a former work of a short sketch of M. Louis' professional life and labours, and is a well-merited eulogium on that conscientious and philosophic physician. The appendix is principally dedicated to a comparison between the results of what may be not improperly termed M. Louis' experiments on the effect of bloodletting, and those obtained by the author in the Massachusett's General Hospital. This comparison being in many points of view interesting, some notice of it may be acceptable to the reader.

It may be not immaterial to state that Dr. Jackson's cases are taken from the registers of the hospital, which appear to be arranged on a plan in the highest degree honorable to the management of the institution; and that all the cases having been recorded previously to the publication of the work of M. Louis, and many of them previously even to that of his original memoir in the Archives Génerales de Médecine, no suspicion can exist of any (unintentional) bias, which might possibly attach to observations made expressly in reference to the question. The basis of Dr. Jackson's calculations are thirty-four cases of pneumonitis, (peri-pneumonia,) a general view of which and of their treatment, with its results, is thrown into a tabular form very convenient for reference. From this comprehensive record he frames tables, calculated, like those of Louis, to show the relative influence of early and late bleedings on the duration of the disease. The material facts furnished by these tables are so distinctly shown by the following commentary of the author, that we feel it unnecessary to transcribe the tables themselves.

"If we take those bled for the first time on the first, second, and third days, together, it will be seen that there were sixteen cases, and that the average period of convalescence was on the $11\frac{13}{16}$, or in decimals 11.81 day. But, omitting case xiii. the result of the fifteen cases will be that convalescence occurred on an average on the $12\frac{1}{3}$, or 12.33 day. If we take those bled for the first time on the fourth day or before, as M. Louis has done, the result will be that, in the twenty-two cases, convalescence took place on an average on the $11\frac{10}{11}$, or 11.90 day. And again omitting xiii., we have as the answer the $12\frac{6}{21}$, or 12.28 day.—As opposed to the foregoing, we may take all those bled for the first time after the fourth day, and we have seven cases in which convalescence took place on an average on the $16\frac{4}{7}$, or 16.57 day. But, omitting cases ix. and xxv., we have five cases, viz. those bled on the fifth, sixth, eighth, and ninth days, in which the average day of convalescence was $13\frac{1}{3}$, or

13.20.—The whole number of those who were bled was twenty-nine; and the average day of convalescence was $13\frac{1}{29}$; or 13.03, or, omitting the three exceptionable cases, for the other twenty-six cases, it was $12\frac{12}{26}$, or 12.46 day.—There remain five cases, in which bloodletting was not employed, except only six leeches in one of them. In these the period of convalescence was on the $14\frac{3}{5}$, or 14.60 day.—We thus see that, so far as the few cases I have furnished go to decide the question, we have shortened the period from the commencement of pneumonitis to the period of convalescence (by bleeding on the first day,) from 14.60 to eleven days. That is, we have diminished the period by about one quarter. If it be said that other remedies were employed, the answer is, that other remedies were employed in all the cases. Next, if we take the least favorable view of the effects, we have diminished the period by about one-tenth." (P. 122-3-4.)

The author subsequently deduces from this passage the inference that the success of bloodletting in his practice is greater than that derived from the same treatment in the hands of M. Louis. We feel, however, some difficulty in reaching the same conclusions, even when allowance is made for the greater mildness of the disease in the cases where bleeding was not employed; for we find that the diminution in the duration, by early in comparison of late bleeding, of both M. Louis' groups of cases of pneumonia, was about one-seventh; whilst Dr. Jackson gains his first average of one-fourth by including a case (the xiii.) which he himself regards as doubtful, and which, if a case of pneumonia at all, was one of this disease supervening on influenza; a category somewhat different from that in which the subjects of M. Louis' observations were found, all of them cases of idiopathic inflammation of the lungs. We find evidence in this appendix, that pneumonia ingrafted on influenza runs its course in a shorter time than the idiopathic disease. Dr. Jackson's researches appear to us to furnish a confirmation of the proposition of Louis, that bloodletting has a happy effect on the progress of pneumonia; that it shortens its duration, but that this influence is much less than has commonly been believed.

It will have been observed by such of our readers as recollect the duration of this disease in M. Louis' practice, that the average period at which convalescence was attained was later by several days than in the cases treated in America. This circumstance is explained (and we believe correctly,) by Dr. Jackson, on the ground that the comfort of the patients is better provided for in the Massachusett's General Hospital than in the larger European establishments, and especially that a higher temperature is preserved in the former than in the Parisian hospitals. Our observation, we admit, has not extended to the American hospitals, but we feel no difficulty in believing that, in respect to the point to which Dr. J. has more particularly adverted, they are superior to those of Paris, which have ever appeared to us to partake largely of that chilling influence which the scarcity and dearness of fuel diffuse over the French metropolis in winter.

We were struck with one remark in this appendix, not because of its novelty, though we acknowledge that it had not before fallen under our observation, but because it accorded with a previously conceived idea of our own: it is in the commentary of the author on his eighth table. No circumstance, he says, exercised so great an influence on the period of convalescence as early admission after the attack: so that it would seem to be less material whether the patients were bled or not, than whether they entered the hospital early or late. The remark is fully borne out by the alleged facts. Twenty entered from the first to the fourth day of the disease, and their average period of recovery was eleven days and a half, or, omitting a case already mentioned as doubtful, nearly twelve days. Twelve entered from the fifth to the sixth day, and the mean period for these was fourteen days and three quarters. Two entered on the fourteenth and one on the fifteenth day, and their average duration was twenty-five days. The being in this building or in that is, of course, not the cause of this difference. It arises from the comparative laxity of the regimen at home, and the rigid discipline and diet of the hospital. Of all our agents for the cure of inflammation, we believe that properly regulated temperature, abstinence—excepting from very mild liquids, and absolute repose, are the most powerful. Were we in our own case compelled to choose between these hygienic means and the most approved therapeutic agents, the adoption of the one necessarily excluding the other, we should select the former. We would not be understood as denying that therapeutic measures possess the power which the general experience of the profession, and the closer investigations of MM. Louis and Jackson, have ascribed to them; but simply as expressing a belief that there has been an error in the estimate of the relative value of the two classes of influences.

There are other points statistically investigated by Louis, which the author tests so far as his cases enable him to do. As to age, his results differ from those of Louis, showing that age had not a retarding influence on the period of recovery. Neither, according to the investigations of Dr. J., had sex, (a subject not examined in the original,) any effect. Vesication of the chest furnished not very decisive results; *but it might be rendered probable that it was useful.* This doubtingly affirmative form of expression, though not the opposite of Louis's sentiment, differs from it. He says that the usefulness of blisters in thoracic inflammation is neither strictly demonstrated, nor even probable. The experience of Dr. J. regarding the effect of venesection on individual symptoms, accords generally with that of the original author.

The principal subsidiary remedies employed in the Massachusett's Hospital were calomel, tartarized antimony, and colchicum. The former was more frequently used in combination with one or other of the two latter than alone. The author's conclusion is, that the difference as to the period of convalescence was so trifling, that it might be regarded as immaterial whether mercurials were given, with or without colchicum or antimony, after bloodletting. In administering tartarized antimony, the method recommended by Odier of Geneva* was pursued. According to this plan, a dose of one-eighth or one-fourth of a grain is first given, and the doses are increased in arithmetical progression until nausea, vomiting, or purging is induced. As soon as any of these symptoms occurs, the dose is reduced to such a quantity as the patient can conveniently bear; or the medicine is suspended till the effect has ceased, and then recommenced in a smaller dose.

Dr. J.'s statistical scrutiny is confined to pneumonia; but he mentions the treatment which he has found most generally successful in erysipelas

* Manuel de Médecine. Paris, 1811.

of the face; a disease on which bloodletting has been found by M. Louis to have but little influence.

"I will venture to add," he says, "that the following has seemed to me the most successful treatment of the disease when seen very early; viz. first, to clear the bowels by a cathartic, and, if specially indicated, the stomach by an emetic; second, to administer the cinchona, or the excellent substitute we now have, the sulphate of quinine. These are given in as large doses as the patient will bear. From twelve to twenty-five grains of the sulphate in twenty-four hours will generally suffice. That the dose is sufficient is known by a buzzing in the ears: when this occurs, the dose may be diminished a little. Third, covering the parts much of the time with a thin linen, which is kept moistened with either diluted alcohol or a solution of acetate of lead. If this treatment is commenced on the very first appearance of the local disease, I think there is a very good chance that the disease will cease to spread, and that the diseased parts will be covered with scales on the fifth day." (P. 102-3.)

This practice coincides in principle with the constitutional treatment recommended generally for erysipelas, traumatic or idiopathic, by Mr. Travers, and other metropolitan authorities in our own country. The nature and treatment of this affection constitute one of the vexed questions of the art, and our impression is, that circumstances extrinsic to the disease, but influencing its character and tendency, have given rise to the diversity of opinion. In certain localities, particularly in large cities, where the constitution is deteriorated by impure air, sedentary pursuits, and a diet at once stimulating and sparingly nutritious, the proclivity to sinking of the vital powers and to local gangrene is great, and in such situations the invigorating plan will be found requisite. But among the rural and more robust population of certain parts of England, the condition of the patient and the influence of remedies have equally indicated a decidedly depletory and antiphlogistic management, including bloodletting; and in such only has safety been found. We know no malady to which the prudent rule of Sydenham, of planning the treatment "perspecto genio morbi," has a more forcible application than to erysipelas. Of the circumstances which render the invigorating method of cure expedient at Boston, we profess our ignorance. It is supposable that some climatorial influence may invest the disease with a character different from that which we have seen it wear in some parts of our own country.

We cannot take leave of Dr. J. without expressing our pleasure at observing the fidelity with which he is endeavouring to carry out the method of Louis for giving us more precise ideas respecting the value of our therapeutic agents. We avail ourselves of the opportunity thus afforded us of again expressing an opinion of the value of this method, to which the professional mind of this country at least is not sufficiently awake,—nay, so far as it is disposed to consider it at all, to which it seems adverse. The questions regarding it which we most frequently hear, are: "To what purpose this counting? Can a man do better than publish all his cases?" We think he can; because cases are scarcely, if ever, read, and because there is not the least chance of the reader being at the trouble of deducing the legitimate inferences from them. This he expects at the hands of the author; and we take leave to recommend the numerical method as well suited to aid the latter in his task.

